# Influence of Light, Temperature, and Nutrient Availability on Growth and Biochemical Composition of *Scenedesmus quadricauda* Cultivated in Municipal Wastewater

**DOI:** 10.3390/microorganisms14010183

**Published:** 2026-01-14

**Authors:** Petras Venckus, Eglė Lastauskienė

**Affiliations:** Life Sciences Center, Institute of Biosciences, Vilnius University, Saulėtekis Ave. 7, LT-10257 Vilnius, Lithuania

**Keywords:** *Scenedesmus*, wastewater treatment, algae biomass production, carbohydrate, protein, lipid

## Abstract

Municipal wastewater contains high amounts of nitrogen (N) and phosphorus (P), as well as other compounds that are harmful to the environment; however, it can also be used as an algae growth medium. In this study locally (Lithuania) isolated algae *Scenedesmus quadricauda* were cultivated in local (Vilnius city) municipal wastewater. Data show that *Scenedesmus* algae can be grown in municipal wastewater as successfully as in Bold’s basal medium for 14 days. Algae cultivation significantly reduced the concentration of organic nitrogen forms and phosphate levels. The nitrogen concentration in wastewater after cultivation was reduced to 8 mg N L^−1^ (up to 89% reduction in total nitrogen concentration). Phosphorus concentration was reduced to 5.4 mg P L^−1^ (up to 86%). The analysis indicates that the optimal temperature for *S. quadricauda* cultivation is 25 °C; temperatures higher or lower than this result in a reduction in algal biomass. A higher amount of light leads to higher yields. No statistically significant differences were found comparing cultivation in BB medium and wastewater under different conditions. The analysis showed that the main factors influencing algae biochemical composition were final total nitrogen concentration and available total nitrogen amount per unit of algae biomass produced, as well as molar N:P ratios. Algae biomass cultivated in wastewater contained a consistent lipid concentration (on average 14.94 ± 2.38%), a lower final total nitrogen concentration, and overall lower total nitrogen availability, leading to higher carbohydrate concentrations (up to 51.10%) and a lower protein content (down to 15.52%). Algae biomass that was cultivated in the BB medium biochemical composition was not dependent on environmental factors and remained consistent (on average 22.89 ± 3.85% carbohydrate, 39.32 ± 3.89% protein, and 13.99 ± 2.21% lipid).

## 1. Introduction

The global economy is expanding, and the demand for energy and other resources is increasing [1]. Algal biomass is one of the most promising alternatives for fossil-fuel-based production and transportation [1,2]. Using microalgae biomass as biofuel or fertilizer does not increase CO_2_ levels in the atmosphere [3,4]. Algae are more productive than plants in terms of biomass accumulation per area [5], and the resulting biomass typically exhibits unique properties [6]. Nevertheless, algae-based products are still more expensive than synthetic fertilizers or fossil fuel sources, and further development of more sustainable technologies is needed. One aspect that could be improved is the use of wastewater instead of an artificial medium for algae cultivation [5]. Various algae species have been proven to be suitable for cultivation in domestic [7,8], agricultural [9,10,11], and various industrial wastewater [2,12,13] settings. Domestic wastewater is known to be suitable for sustaining algal growth [14]. Usually, it is considered waste; therefore, it is a very inexpensive alternative to artificial algae medium [7]. Its chemical composition directly affects the growth rate and biochemical composition of algae biomass [8]. Nutrients responsible for eutrophication (nitrogen and phosphorus compounds) could be assimilated by growing various algae species [9,10]. The assimilation rates of these nutrients depend on various factors, including the environmental conditions [15], type of algae used [16], and composition of the wastewater [11]. Obtained biomass can be utilized as a feedstock, biofertilizer, or biofuel [12,13,16]. However, municipal wastewater is often characterized by strong variability and elemental imbalance, particularly in N:P ratios, which can slow microalgal growth and metabolic performance.

Microalga physiology is strongly influenced by elemental stoichiometry. The Redfield ratio (N:P = 16:1) and its freshwater adaptations provide a conceptual framework for diagnosing nutrient limitation and predicting biochemical allocation patterns [17,18]. Deviations from optimal N:P ratios can alter nutrient uptake kinetics and induce major changes in cellular composition [19]. This imbalance can also affect wastewater treatment efficiency [10,20].

Understanding algae physiology and the influence of various environmental factors on biomass growth and biochemical composition is critical [6]. Various factors such as the presence of bacteria, heavy metals, or other toxic compounds can hinder algae growth [15,21]. Other factors such as wastewater turbidity or the presence of solid particles are more prominent in large-scale cultivation [22]. Species of the genus *Scenedesmus* have been widely reported as efficient candidates for nutrient removal and biomass production in wastewater systems, including municipal wastewater (e.g., *Scenedesmus quadricauda*, *S. obliquus*, *Scenedesmus* sp.), with high removal efficiencies for nitrogen and phosphorus. However, most studies either use fixed cultivation conditions or do not systematically combine gradients of light and temperature under real municipal wastewater conditions, indicating a lack of comprehensive data on indigenous strains in controlled multi-factor environments [23,24,25,26], especially the influence of temperature and light conditions on various algae species. Additionally, indigenous algae are already acclimated to local climatic conditions and may have natural advantages over similar species cultures isolated from different environmental conditions [27]. Thus, indigenous strains may be more productive under certain conditions [24], and these conditions significantly impact the biochemical composition of biomass [27].

This study utilized the indigenous *S. quadricauda* strain isolated from a lake in Lithuania. We investigated the impact of environmental factors (amount of light and temperature) and the chemical composition of the media (amount of total nitrogen and phosphorus) on biomass growth and biochemical composition. We used local (Vilnius city) municipal wastewater and Bold’s basal medium to determine the possibility of using wastewater as a medium for the examined strain.

## 2. Materials and Methods

### 2.1. Organism and Culture Conditions

We used an isolate of the species *Scenedesmus quadricauda* (Turpin) Brébisson 1835 isolated from Lithuania’s Lake Babrukas (54.623911 24.93409). Inocula were cultivated in Bold’s basal media (BBM) [28] in 400 mL of medium in 1 L Erlenmeyer flasks using artificial illumination (6000 K 25 W LED tubes) with a light periodization of 16 h of light and 8 h of darkness and a light intensity of 56 µmol m^2^ s^−1^, which corresponded to 700 kJ m^−2^ day^−1^. The municipal wastewater used in the experiments was collected from the Vilnius municipal wastewater treatment plant after the primary treatment (sedimentation ponds) one day before the start of experimental trials. Before use, wastewater was filtered using filtering paper to separate the liquid and solid phases, and samples were preserved for the determination of nitrogen and phosphorus. Bold’s basal (BB) medium was used as a control.

Experiments were performed in a cultivation chamber with controlled temperature and under artificial light provided by white fluorescent tubes (6000 K; 25 W). Light periodization was 16:8 h of light and darkness, starting the experiment in the light period. Three different daily light amounts 223 kJ m^2^ day^−1^, 700 kJ m^2^ day^−1^, and 1165 kJ m^2^ day^−1^ were used (light intensity of 18 µmol m^2^ s^−1^, 56 µmol m^2^ s^−1^, and 93 µmol m^2^ s^−1^, respectively. Four different temperatures (15 °C, 20 °C, 25 °C, 30 °C) were tested (two biological replicates). Air was supplied from the bottom of the flask to mix cultures and increase the gas exchange rate between the air and the culture. pH was not controlled; it was only monitored every 2 days. At the start of the experiment, 1 L flasks were filled with 350 mL of wastewater or medium (for the control) and 50 mL of inoculum, providing an initial biomass concentration to 0.2 g dry weight L^−1^. Samples of medium and inoculum were taken for the determination of nitrogen and phosphorus. Cultures were performed in batches for 14 days. An amount of 30 mL of culture was taken from each flask every two days to determine dry weights. An amount of 30 mL of wastewater or medium was added to compensate for the loss of culture volume. The loss of volume due to evaporation was compensated for by adding deionized water. After 14 days, the culture samples were centrifuged, and the algae biomass was dried for 2 days at 45 °C and then preserved at −20 °C for further biochemical analyses. Medium and wastewater samples were also preserved for the determination of residual nitrogen and phosphorus.

### 2.2. Analytical Procedures

Culture growth was estimated by measuring dry biomass concentration. Dry weight was determined by centrifuging a sample, drying it at 70 °C for at least three hours, and weighing [29]. Total phosphorus concentration was determined using the ammonium molybdate spectrometric method (ISO 6878:2004 [30]), and total nitrogen was determined by high-temperature catalytic oxidative combustion coupled with chemiluminescence detection using TOC-Lcsh with a TNM-1 unit (Shimadzu Corporation, Kyoto, Japan) (ISO 20236:2024 [31]). The lyophilized biomass was analyzed for carbohydrates using the phenol–sulfuric acid method [19] and protein using Lowry’s method [32]. Lipid content was determined spectrophotometrically after carbonization of the material extracted with a 2:1 methanol/chloroform solution [33,34]. Tripalmitin (Sigma-Aldrich, Milan, Italy) was used as a standard [35].

### 2.3. Statistical Analysis

We used R (version 4.7.0) (R Core Team, 2024) for statistical analysis. The Shapiro–Wilk test was used to test normality, and Bartlett’s test was used to test the equality of variances. The Wilcoxon matched-pairs signed-rank test was used to determine changes in nitrogen and phosphorus consumption in the BB medium and wastewater. Multiple linear regression was used to describe nitrogen consumption in the BB medium. Two-way ANOVA with Tukey’s pairwise comparison test was used to determine the influence of light and temperature on algae culture growth. A *t*-test was used to determine the growth differences between the medium and the wastewater in each treatment. Spearman correlation analysis was used to determine if there is a correlation between nitrogen and phosphorus consumption and growth. Regression analysis was used to assess the impact of the final nitrogen concentration in the medium (BB medium and wastewater) on the biochemical composition of the biomass (exponential curves) and the impact of overall production on biochemical composition (linear regression). The Kruskal–Wallis test with Dunn’s pairwise comparison test was used to determine the effect of temperature and light conditions on the biochemical composition of the biomass cultivated in the BB medium and to investigate the influence of light and temperature on nitrogen and phosphorus consumption. The significance level for all tests performed was *p* < 0.05.

## 3. Results

### 3.1. Effect of Environmental Conditions on Phosphorus and Nitrogen Concentration Changes

**Figure 1:**
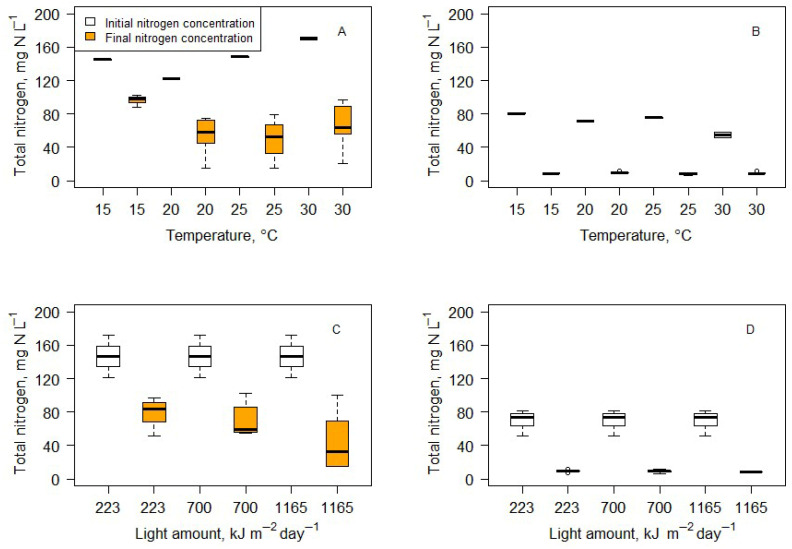
Initial and final total nitrogen concentrations under different temperature conditions in medium (**A**) and wastewater (**B**) and under different light conditions in medium (**C**) and wastewater (**D**).

**Figure 2:**
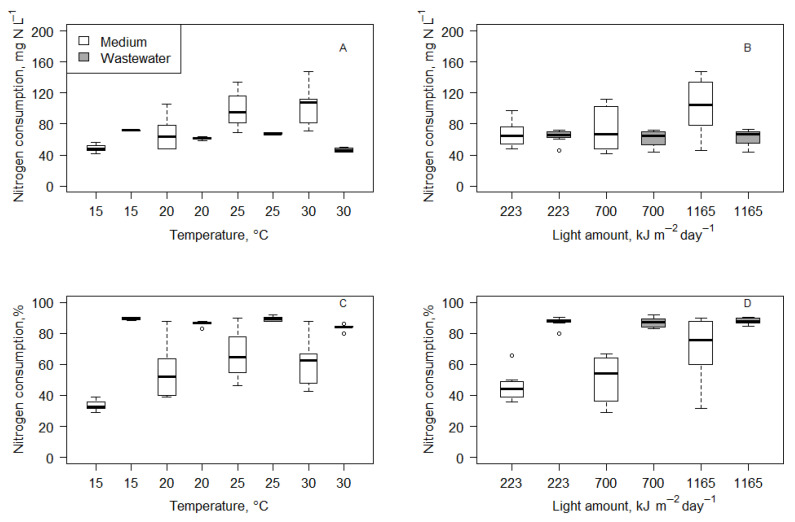
Nitrogen consumption (mg N L^−1^) after a 14-day cultivation of *Scenedesmus quadricauda* in BB medium and wastewater under different temperature (**A**) and light (**B**) conditions. Percentage of total removed nitrogen after 14 days of cultivation in medium and wastewater under different temperature (**C**) and light (**D**) conditions.

The change in nitrogen concentration in medium and wastewater after 14 days of cultivation is shown in Figure 1. The initial median nitrogen concentration in the medium was 146 mg N L^−1^ minimum of 120.9 mg N L^−1^ and maximum of 171.4 mg N L^−1^). The final median nitrogen concentration was 68.9 mg N L ^−1^ (maximum of 102.8 mg N L^−1^ and minimum of 14.8 mg N L^−1^). Wastewater initial nitrogen concentration was 73.8 mg N L^−1^ (the maximum was 80.9 mg N L^−1^ and the minimum was 51.4 mg N L^−1^). The final median nitrogen concentration was 9.0 mg N L ^−1^ (maximum of 12.0 mg N L^−1^ and minimum of 6.3 mg N L^−1^). The Wilcoxon matched-pairs signed-rank test results showed that nitrogen concentration was significantly reduced in all treatments in medium and wastewater (Figure 1 and Figure 2).

There was almost no variation in final nitrogen concentration in wastewater in different treatments (no statistically significant variation). The final nitrogen concentration in wastewater was 8.89 ± 1.23 mg N L^−1^, or a 87.25 ± 2.84% reduction compared to the initial nitrogen concentration. The data suggest that the cells could not uptake nitrogen if the concentration dropped below a specific limit (~8 mg N L^−1^). However, no statistical analysis methods can be used to determine how light and temperature conditions influence nitrogen consumption.

The results of multiple linear regression showed that higher light amounts and higher temperatures significantly increased nitrogen consumption rates in BB medium (Formula (1)).

(1) $$Nitrogen\;consumption\;\mathrm{mg}\;L^{-1}=0.006\;light\;{\mathrm{kJ}\;m}^{-2}{\mathrm{day}}^{-1}\times 3.846\;{}^{\circ}C.$$

Formula (1). Nitrogen consumption depends on the light and temperature conditions during the 14 days of *S. quadricauda* cultivation in BB medium (*p* < 0.05; R^2^ = 0.71).

Neither temperature nor amount of light alone had a statistically significant influence on nitrogen consumption.

**Figure 3:**
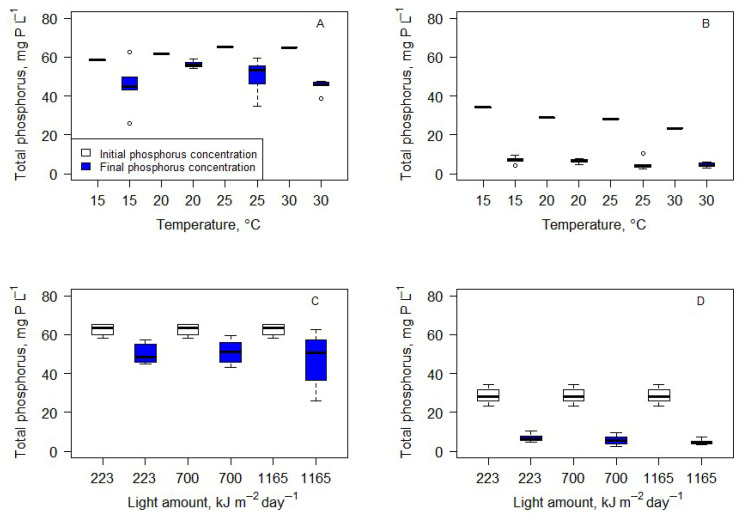
Initial and final total phosphorus concentrations under different temperature conditions in medium (**A**) and wastewater (**B**) and under different light conditions in medium (**C**) and wastewater (**D**).

As well as changes in nitrogen concentration, changes in phosphorus (in the form of orthophosphate ions) were observed (Figure 3). The initial concentration of phosphorus in the medium was 63.45 mg P L^−1^ (maximum of 65.34 mg P L^−1^ and minimum of 58.31 mg P L^−1^). After 14 days of cultivation, the phosphorus concentration dropped to 48.64 mg P L^−1^ (maximum of 59.50 mg P L^−1^ and minimum of 26.09 mg P L^−1^). Meanwhile, wastewater had half the initial concentration of phosphorus, 28.18 mg P L^−1^ (the maximum was 34.46 mg P L^−1^ and the minimum was 23.13 mg P L^−1^). After the experiment, the phosphorus concentrations dropped to 5.37 mg P L^−1^ (maximum of 10.54 mg P L^−1^ and minimum of 2.66 mg P L^−1^). The Wilcoxon matched-pairs signed-rank test results showed that phosphorus concentrations significantly dropped in all treatments.

**Figure 4:**
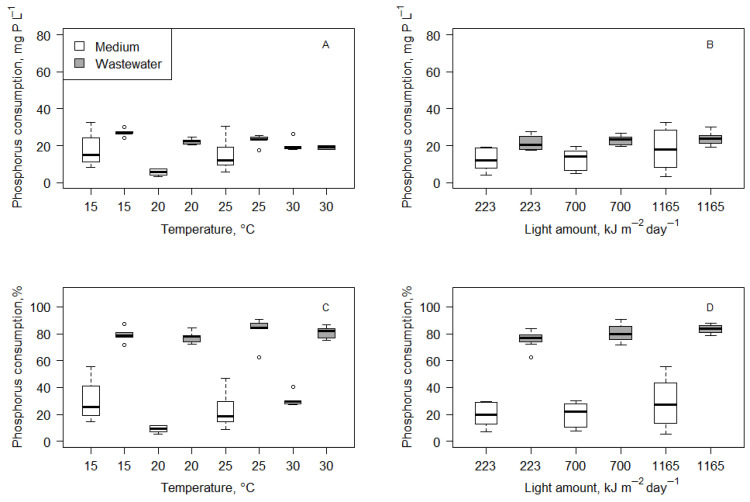
Phosphorus consumption (mg P L^−1^) after a 14-day cultivation of *Scenedesmus quadricauda* in BB medium and wastewater under different temperature (**A**) and light (**B**) conditions. Percentage of total removed phosphorus after 14 days of cultivation in medium and wastewater under different temperature (**C**) and light (**D**) conditions.

Statistical analysis (regression and Kruskal–Wallis test) showed that environmental factors (light amount and temperature) did not have a significant impact on phosphorus consumption (*p* > 0.05) in medium and wastewater (Figure 4). However, it has to be mentioned that phosphorus consumption was higher in wastewater than medium in almost all treatments. In the medium, it was only 14.27 ± 8.42 mg P L^−1^, which was, on average, a 22.58 ± 13.39% reduction from the initial phosphorus concentration. In wastewater, it was 22.82 ± 3.49 mg P L^−1^, which was, on average, a 80.00 ± 6.39% reduction from the initial phosphorus concentration. Further analysis of combined data from wastewater and medium cultivation showed that a phosphorus consumption of mg P L^−1^ had a moderate negative correlation to initial phosphorus concentration (Spearman r = −0.32; *p* < 0.05). Also, similarly to nitrogen concentration, phosphorus concentration did not drop below a specific value, which was about 2.7 mg P L^−1^.

### 3.2. Stoichiometric Conditions

Based on molar conversions of total nitrogen and total phosphorus concentrations, both BB medium and wastewater were characterized by relatively low N:P ratios. In BB medium, the mean initial molar N:P ratio was 5.17, which decreased to 2.90 by the end of cultivation. Similarly, municipal wastewater exhibited a mean initial N:P ratio of 5.47, which declined to 3.78 after cultivation. In both media, the reduction in N:P ratio resulted primarily from a stronger decrease in nitrogen concentration relative to phosphorus. Final N:P ratios in BB frequently approached values close to 3, whereas wastewater maintained slightly higher final ratios, although they were still well below the classical Redfield ratio of 16:1. However, BB medium had much higher absolute concentrations of nitrogen and phosphorus compounds.

### 3.3. Effect of Environmental Conditions on Culture Biomass Production

**Figure 5:**
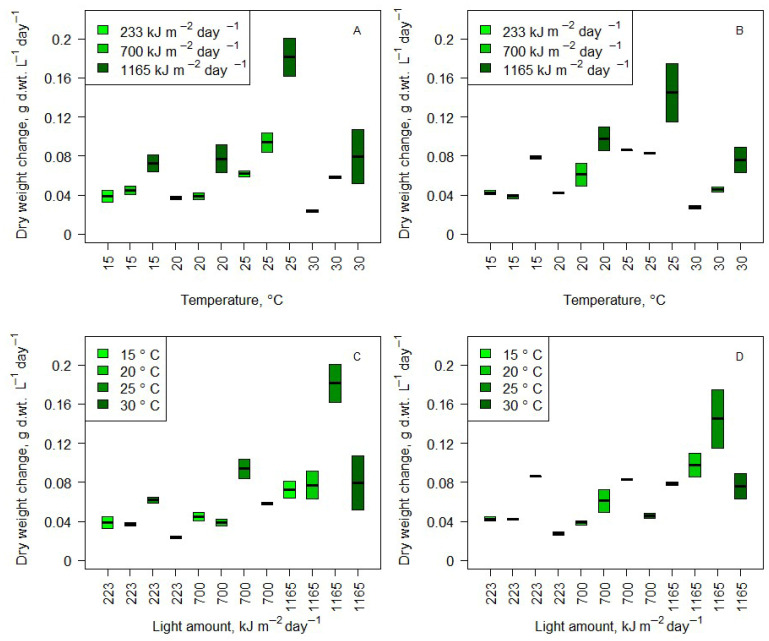
Daily dry weight changes of *Scenedesmus quadricauda* algae under different temperature conditions in medium (**A**) and wastewater (**B**) and under different light conditions in medium (**C**) and wastewater (**D**).

Production data were acquired by measuring the dry weight every two days during the experiment. The daily production dependences from light and temperature conditions in medium and wastewater are presented in Figure 5. To determine the influence of temperature, light, and medium (medium and wastewater), two-way ANOVA with Tukey’s pairwise comparison test was used. Results showed that the light conditions and temperature had a significant effect on growth. In the medium, 40.5% of the variation was determined by the light conditions and 38.3% by the temperature conditions. Similar results were observed in wastewater treatments, with 43.5% of variation dependent on the light conditions and 42.7% on the temperature conditions. The influence of light was linear (higher light amount contributed to higher growth linearly) (Figure 5A,B). However, the temperature influence was not linear. The highest growth was observed at 25 °C (Figure 5C,D). Results did not show significant differences between growth in cultures of 15 °C (higher light did not significantly affect growth). In 20 °C and 30 °C treatments, a statistically significant growth increase was observed compared with cultures with 223 kJ m^2^ day^−1^ light, amounting to 1165 kJ m^2^ day^−1^. Light conditions had the highest impact in cultures cultivated at 25 °C conditions. A significant increase in growth was observed comparing 223 kJ m^2^ day^−1^ to 1165 kJ m^2^ day^−1^, as well as 700 kJ m^2^ day^−1^ to 1165 kJ m^2^ day^−1^ cultures (in medium and wastewater). *t*-tests were used to determine the growth differences in every treatment between medium and wastewater. No statistically significant differences were found.

**Figure 6:**
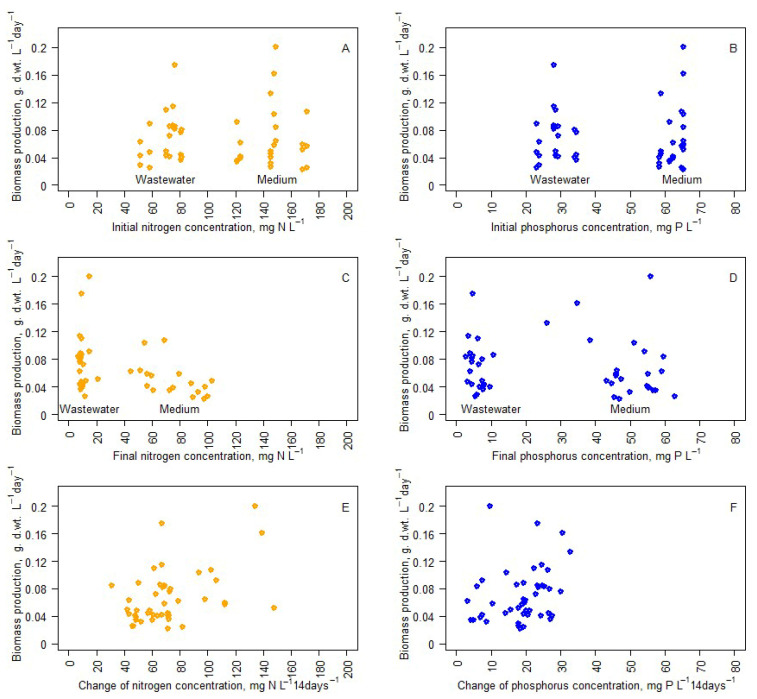
Daily dry weight changes depend on initial total nitrogen (**A**) and final total nitrogen (**C**) concentrations, as well as initial total phosphorus (**B**) and final total phosphorus (**D**) concentrations. Daily dry weight changes and nitrogen (**E**) and phosphorus (**F**) consumption.

Spearman correlation analysis was used to determine the effect of initial nitrogen and phosphorus (Figure 6A,B), final nitrogen and phosphorus (Figure 6C,D), and nitrogen and phosphorus consumption (Figure 6E,F) on growth. No correlations were found between any of the aforementioned factors and growth. Therefore, these factors influenced the biochemical composition of the biomass.

### 3.4. Effect of Environmental Conditions on Culture Biomass Biochemical Composition

**Figure 7:**
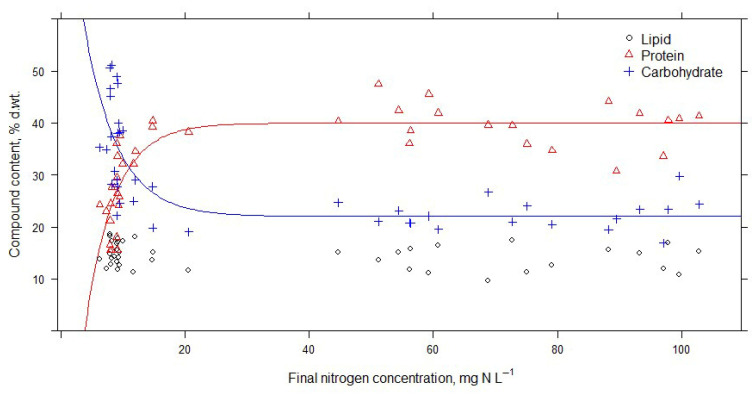
Carbohydrate, protein, and lipid content in the biomass depends on final total nitrogen concentration. Lines represent best-fit curves for protein (red line) and carbohydrate (blue line) contents (Formulas (2) and (3)).

Biochemical analysis data showed very different results and dependencies compared to production. The variation in final nitrogen concentration can explain changes in carbohydrate and protein concentration (Figure 7). Formulas (2) and (3) represent best-fit curves (one-phase decay with least squares fit) to protein (Formula (2)) and carbohydrate (Formula (3)) content dependency on final nitrogen concentration. A visual representation of these curves can be seen in Figure 7.

(2) Pt=−91.16×e(−0.21×FinN)+39.97

Formula (2). Protein content in the biomass (*Pt*) depends on the final nitrogen concentration (mg N L^−1^) in the medium (*FinN*). R^2^ = 0.68.

(3) Ch=75.25×e(−0.18×FinN)+22.01

Formula (3). Carbohydrate content in the biomass (*Ch*) dependency fromfinal nitrogen concentration (mg N L^−1^) in the medium (*FinN*). R^2^ = 0.58.

Lipid content showed no dependence on final nitrogen concentration. (Linear regression *p* > 0.05).

**Figure 8:**
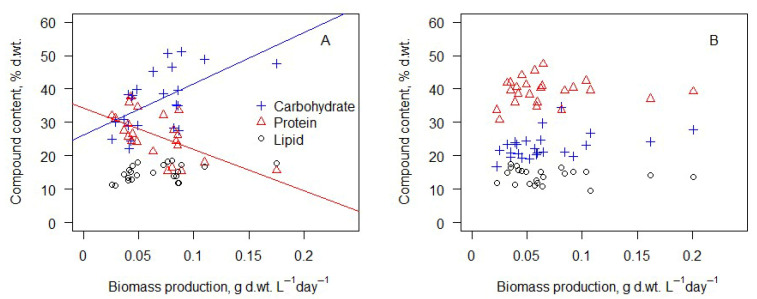
Carbohydrate, protein, and lipid content in the biomass depends on daily biomass production in wastewater (**A**) and BB medium (**B**). Lines represent statistically significant linear regression curves for carbohydrate (blue) and protein (red) content in biomass cultivated in wastewater.

Linear regression analysis was performed to determine biomass production’s influence on the biomass’s biochemical composition. No statistically significant biochemical compound content dependence from biomass production was found by analyzing biomass cultivated in BB medium (Figure 8B). Analysis of biomass that was grown in wastewater showed that higher production corresponded to significantly higher carbohydrate (Formula (4)) content and lower protein (Formula (5)) content (Figure 8).

(4) Ch=153.49 × Production+26.22

Formula (4). Carbohydrate content in the biomass (*Ch*) depends on daily biomass production (g dry weight L^−1^ day^−1^) in wastewater (*Production*). R^2^ = 0.39

(5) Pt=−123.36 × Production+34.27

Formula (5). Protein content in the biomass (*Pt*) depends on daily biomass production (g dry weight L^−1^ day^−1^) in wastewater (*Production*). R^2^ = 0.37

**Figure 9:**
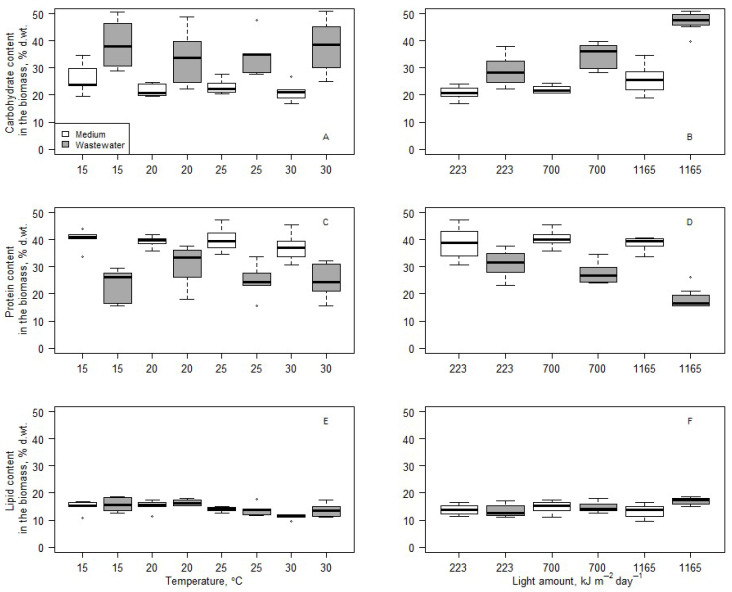
Carbohydrate content in the biomass’ dependence on temperature (**A**) and light (**B**) conditions. Protein content in the biomass’ dependence on temperature (**C**) and light (**D**) conditions. Lipid content in the biomass’ dependence on temperature (**E**) and light (**F**) conditions.

Figure 9 represents biochemical changes in the biomass after 14 days of cultivation depending on the light and temperature conditions. Due to nitrogen deprivation in cultures cultivated in wastewater (Figure 7), statistical analysis (Kruskall–Wallis test) was performed only to determine the influence of light and temperature on algae biochemical composition in cultures involving BB medium. The test showed no statistically significant differences between carbohydrate and protein contents in the biomass cultivated at various temperatures and light conditions. Only the lipid content was significantly lower in the biomass cultivated at 30 °C compared to 15 °C and 20 °C. Changes in the amount of light did not influence the lipid content in the biomass.

## 4. Discussion

### 4.1. Nitrogen and Phosphorus Removal

The results of this study indicate that *Scenedesmus quadricauda* has significant potential for nitrogen and phosphorus removal from municipal wastewater, demonstrating its effectiveness as a sustainable bioremediation tool. Algae from the genus *Scenedesmus* demonstrated good nutrient (N and P) removal rates under different conditions and when cultivated in different types of wastewater [14,36]. The observed reductions in nitrogen and phosphorus concentrations confirm that the algae actively assimilated these nutrients, although the environmental conditions influenced their consumption rates. Other studies have shown that the nutrient consumption level is dependent on initial nutrient concentration as well as the environmental conditions [36].

The findings reveal that nitrogen consumption was strongly influenced by light intensity and temperature in BB medium but not in wastewater. This suggests that while *S. quadricauda* benefits from optimal environmental conditions in synthetic media, its ability to remove nitrogen from actual wastewater is more stable and less dependent on external factors. The observed threshold of ~8 mg N L^−1^, below which nitrogen uptake ceased, may indicate a physiological limitation point beyond which further uptake does not occur. Similar thresholds were observed in different studies. *Neochloris oleoabundans* did not reduce nitrogen concentration to levels lower than 8 mg N L^−1^ and various other algae have had thresholds lower than 1–2 mg N L^−1^ [14]. This indicates that our measured nitrogen uptake level in wastewater of ~5 mg N L^−1^ day^−1^ was not the highest possible uptake speed and was limited by the low initial nitrogen concentrations in wastewater. Our obtained nitrogen removal rate in wastewater of about ~87% is very similar to the rates measured in other studies (67.4–99.9%) [37].

Similarly, phosphorus removal was more pronounced in wastewater than in BB medium, with an average reduction of 80% compared to 22.58% in BB medium, which corresponds to average reductions of 22.3 mg P L^−1^ and 14.3 mg P L^−1^. This suggests that wastewater contains additional factors that enhance phosphorus uptake, possibly due to the presence of organic matter or microbial interactions. However, statistical analyses indicated no significant influence of temperature or light conditions on phosphorus removal, suggesting that its uptake is more dependent on initial phosphorus concentrations than environmental conditions. This collaborates the findings of Chen et al., which showed that phosphorus removal is dependent on the initial phosphorus concentration as well as the algae used [36]. Stoichiometric analysis revealed that molar N:P ratios provided critical insight into nutrient uptake dynamics that could not be explained by absolute concentrations alone. Initial N:P ratios in wastewater were substantially lower than the classical Redfield ratio, indicating potential nitrogen limitation from the onset of cultivation. Also, absolute values in mg N or P per liter were much lower compared to BB medium. During the 14-day cultivation period, N:P ratios declined further in both media, with a markedly stronger decrease observed in wastewater, where final N:P ratios frequently fell below 4 and, in some cases, approached values < 2.

These results indicate preferential nitrogen removal relative to phosphorus and suggest that nitrogen became the primary limiting nutrient, particularly in wastewater. Phosphorus concentrations declined more slowly, consistent with luxury uptake and intracellular storage mechanisms reported for freshwater microalgae [18,35]. The stoichiometric framework also explains why nitrogen consumption responded to light and temperature in BB medium but not in wastewater. In BB medium, nutrient availability remained sufficiently high to allow environmental drivers to modulate nitrogen uptake. In contrast, rapid nitrogen depletion and persistently low N:P ratios in wastewater imposed a strong physiological constraint on nitrogen assimilation, effectively overriding the influence of light and temperature. Similar decoupling of nutrient uptake from environmental drivers under stoichiometric limitations has been reported in recent wastewater cultivation studies [10,20].

Additionally, other studies have shown that other factors like wastewater turbidity and dissolved organic matter can reduce effective light penetration, potentially diminishing the influence of external light intensity. Finally, interactions with indigenous bacterial communities may have enhanced nutrient transformation and availability through mineralization processes, contributing to stable phosphorus removal even under nitrogen-limited conditions [11].

### 4.2. Biomass Production

Both light and temperature significantly influenced biomass production, with the highest yields observed at 25 °C and under high light conditions (1165 kJ m^2^ day^−1^). The temperature dependence was not linear, with suboptimal growth at 15 °C and 30 °C, highlighting the importance of maintaining a moderate thermal range for optimal cultivation. There was a lack of significant differences between growth in BB medium and wastewater. Also, data show that a higher amount of light has a more significant positive effect on biomass accumulation only in optimal temperature conditions. Interestingly, the slowest growth was observed in high (30 °C) temperature and low light (233 kJ m^2^ day^−1^). This was observed during all repetitions in cultures cultivated in BB medium, as well as in wastewater. Growth was not slowed by nitrogen or phosphorus deprivation, and growth patterns remained very similar in BB medium and wastewater. *S. quadricauda* growth did not slow even in very unfavorable stoichiometric conditions; instead, changes in its biochemical composition were observed, mostly changes in its carbohydrate and protein ratio. This further emphasizes the adaptability of *S. quadricauda* to real-world wastewater conditions and its potential to be used to obtain biomass of a desired biochemical composition.

### 4.3. Biomass Biochemical Composition

The biochemical composition of the biomass was largely influenced by nitrogen availability rather than environmental factors. The observed stoichiometric shifts provide an explanation for the contrasting biochemical responses between biomass grown in BB medium and wastewater. Proteins, as nitrogen-rich macromolecules, declined sharply with decreasing nitrogen availability, whereas the carbohydrate content increased under nitrogen-depleted conditions. The highest daily protein production was obtained at optimal growth conditions (25 °C and 1165 kJ m^2^ day^−1^). Biomass cultivated in low-light (223 kJ m^2^ day^−1^) conditions was slightly richer in proteins, but the amount of daily biomass was very low compared to high-light conditions. Lower nitrogen levels led to greater carbohydrate accumulation. There is obvious correlation between nitrogen shortage and carbohydrate content and by extension between the seriousness of the shortage and the time algae spent in nitrogen shortage conditions and carbohydrate content in the biomass. This was observed only in wastewater, where the nitrogen content was too low to sustain algae growth without changes in biomass biochemical composition, as was observed in the biomass cultivated in BB medium, which is very rich in nitrogen. This aligns with previous studies indicating that nitrogen limitation triggers carbon storage mechanisms in microalgae and fixed carbon is diverted to nitrogen-poor storage compounds [38]. The rapid decline of nitrogen concentrations in wastewater and the stabilization near ~8 mg N L^−1^ indicate the onset of nitrogen limitation, which is known to downregulate nitrogen assimilation pathways, including the GS–GOGAT system, and restrict protein biosynthesis [19,38]. The lipid content was not significantly affected by nitrogen concentration but was lower at 30 °C, indicating possible metabolic shifts under thermal stress. Under moderate nitrogen limitation and short-term batch cultivation, carbon overflow was preferentially directed toward carbohydrates rather than lipids, as reported for *Scenedesmus* and related freshwater species [14,16]. Lipid contents of 10–20% of total dry weight are on the lower side compared to other studies, which indicates that this culture is not suitable for lipid-rich algae biomass production [14].

### 4.4. Final Considerations

These findings have important implications for large-scale wastewater treatment applications. The high nutrient removal efficiencies and robust biomass production suggest that *S. quadricauda* could be considered and integrated into existing wastewater treatment processes as a sustainable alternative to conventional treatment methods. The ability to generate biomass with varying biochemical compositions further enhances its value for bio-product production.

Despite promising results, this study has certain limitations. The study period was limited to 14 days, which may not capture all short-term or long-term performance variations. Additionally, external microbial communities present in wastewater could have influenced nutrient uptake dynamics, warranting further investigation. Parameters such as organic nutrient fractions, pH fluctuations, turbidity, dissolved oxygen dynamics, and microbial interactions were not explicitly controlled for and may contribute to variability in nutrient utilization efficiency. Future studies combining the physicochemical characterization of wastewater with metabolic or transcriptomic analyses would provide deeper insight into the mechanisms governing nitrogen and phosphorus assimilation under real wastewater conditions and explore the scalability of this approach in pilot-scale wastewater treatment systems, investigate potential co-cultivation strategies with other microorganisms, and assess the economic feasibility of large-scale implementation.

## 5. Conclusions

*Scenedesmus quadricauda* demonstrated strong potential for municipal wastewater treatment, achieving high nitrogen and phosphorus removal efficiencies regardless of the environmental conditions. Nitrogen uptake depended on light and temperature in BB medium but remained consistently high in wastewater, with both nutrients showing threshold concentrations below which further assimilation did not occur. These findings emphasize the importance of stoichiometric considerations in the design and optimization of microalgae-based wastewater treatment systems. Declining N:P ratios under wastewater conditions induced nitrogen limitation, triggering metabolic reallocation toward carbohydrate accumulation at the expense of protein synthesis, while the lipid content remained relatively stable. The N:P ratio did not have any significant impact on algae grown in BB medium due to its high absolute concentrations of nitrogen and phosphorus compounds.

Biomass production was significantly influenced by light and temperature, with optimal growth at 25 °C and high light intensity. While growth patterns were similar in BB medium and wastewater, nitrogen limitation in wastewater led to marked changes in biochemical composition, increasing the carbohydrate content and reducing protein levels. Lipid content was largely stable except under thermal stress at 30 °C.

Overall, the species shows promise for integration into sustainable wastewater treatment systems, combining effective nutrient removal with the ability to generate biomass of variable biochemical compositions suitable for different biotechnological applications.

## Data Availability

The raw data supporting the conclusions of this article will be made available by the authors on request.
